# Diabetes mellitus impairs bone regeneration and biomechanics

**DOI:** 10.1186/s13018-023-03644-5

**Published:** 2023-03-06

**Authors:** Feiyu Cai, Yanshi Liu, Kai Liu, Ruomei Zhao, Wenjiao Chen, Aihemaitijiang Yusufu, Yi Liu

**Affiliations:** 1 Department of Burns and Plastic Surgery and Wound Repair Surgery, The Lanzhou University Second Hospital, Lanzhou, Gansu China; 2 Department of Trauma and Micro Reconstructive Surgery, The First Affiliated Hospital of Xinjiang Medical University, Ürümqi, Xinjiang China; 3 Department of Orthopaedics, The Affiliated Hospital of Southwest Medical University, Luzhou, Sichuan China

**Keywords:** Angiogenesis, Biomechanics, Bone regeneration, Diabetes

## Abstract

**Background:**

With the rise of high-calorie diets and the aging of populations, the incidence of diabetes was increased dramatically in the world and the number of people with diabetes was predicted to rise to 600 million by 2045. Numerous studies have confirmed that several organ systems, including the skeletal system, are seriously affected by diabetes. In that study, the bone regeneration and the biomechanics of the newly regenerated bone were investigated in diabetic rats, which may provide a supplement for previous studies.

**Methods:**

A total of 40 SD rats were randomly divided into the type 2 diabetes mellitus (T2DM) group (*n* = 20) and the control group (*n* = 20). Beyond that high fat diet and streptozotocin (STZ) were jointly used in the T2DM group, there were no differences between the two groups in terms of treatment conditions. Distraction osteogenesis was used in all animals for the next experimental observation. The evaluation criterion of the regenerated bone was based on radioscopy (once a week), micro-computed tomography (CT), general morphology, biomechanics (including ultimate load, modulus of elasticity, energy to failure, and stiffness), histomorphometry (including von Kossa, Masson trichrome, Goldner trichrome, and safranin O staining), and immunohistochemistry.

**Results:**

All rats in the T2DM group with fasting glucose levels (FGL, > 16.7 mmol/L) were allowed to complete the following experiments. The results showed that rats with T2DM have a higher body weight (549.01 g ± 31.34 g) than rats in the control group (488.60 g ± 33.60 g) at the end of observation. Additionally, compared to the control group, slower bone regeneration in the distracted segments was observed in the T2DM group according to radiography, micro-CT, general morphology, and histomorphometry. Furthermore, a biomechanical test showed that there was a worse ultimate load (31.01 ± 3.39%), modulus of elasticity (34.44 ± 5.06%), energy to failure (27.42 ± 5.87%), and stiffness (34.55 ± 7.66%) than the control group (45.85 ± 7.61%, 54.38 ± 9.33%, 59.41 ± 10.96%, and 54.07 ± 9.30%, respectively). Furthermore, the decreased expressions of hypoxia-inducible factor 1α (HIF-1α) and vascular endothelial growth factor (VEGF) were presented in T2DM group by immunohistochemistry.

**Conclusion:**

The present study demonstrated that diabetes mellitus impairs bone regeneration and biomechanics in newly regenerated bone, a phenomenon that might be related to oxidative stress and poor angiogenesis brought on by the disease.

## Background

With the rise of high-calorie diets and the aging of populations, the incidence of diabetes was increased dramatically in the world. The World Health Organization (WHO) estimated in 1980 that 108 million people lived with diabetes, and the number increased fourfold by 2014 [1]. A study has reported that the number of people with diabetes was predicted to rise to 693 million by 2045 [2]. It is worth noting that various complications caused by diabetes posed a huge challenge to modern medical care, including urinary, circulatory, digestive, and motor systems. Previous studies have demonstrated that hyperglycemia has a negative influence on the skeletal system [3, 4]. Compared to healthy people, patients with diabetes are more likely to suffer a bone fracture due to low bone mineral density (BMD) [5]. Additionally, patients with type 2 diabetes mellitus (T2DM) often suffer various osteopathy, which may be associated with increased production of protein kinase C (PKC), advanced glycation end products (AGEs), and reactive oxygen species (ROS) under a hyperglycemic environment [6, 7]. Furthermore, hyperglycemia can induce chronic kidney disease that may damage normal bone metabolism, decrease BMD, and induce bone fracture [8, 9]. These studies have primarily investigated how diabetes can cause fractures and the possible mechanisms involved. In the present study, a hypothesis we propose is that diabetes may delay bone regeneration after fractures and decrease biomechanics.

To test this hypothesis, the distraction osteogenesis (DO) technique was used to establish an animal model that will enable us to observe bone regeneration more clearly following trauma. DO is a surgical technique that stimulates bone tissue regeneration by stretching tension forces on severed bone tissue [10]. DO is widely used in experimental research of bone regeneration and clinical treatment that includes limb discrepancy, bone nonunion, bone infection, bone defect, and malformation [11–15]. In this study, a series of observations were conducted to assess the impact of diabetes on bone regeneration and biomechanics, including radioscopy, micro-computed tomography (CT), general morphology, biomechanics, histomorphometry, and immunohistochemistry.

## Materials and methods

### Animals

Forty male Sprague–Dawley (SD) rats (5 weeks old) were used in this study and randomly divided into the T2DM group (*n* = 20) and the control group (*n* = 20) in accordance with the random number table. Animals were raised at a temperature of 20–25 °C and a humidity of 50–60% with free access to water and a pelleted diet. All experimental procedures were approved by the Animal Ethics Committee of Xinjiang medical university (IACUC-202003318-82).

In this study, high-fat-diet (HFD, 60 kcal% fat, Beijing Boaigang Biotechnology Co. Ltd, Beijing, China) and streptozotocin (STZ, Sigma, USA) intraperitoneal injections were used in the T2DM group. After the rats received an HFD for four weeks, the STZ (30 mg/kg) was used to generate a diabetic model according to a previous study [16]. Tail venous blood was collected to evaluate a standardized diabetic model by using the fasting glucose levels (FGL) at 1 and 2 weeks following STZ injection. Rats with applicable FGL (> 16.7 mmol/L) were included in the next experiments. In order to stabilize the diabetic model, HFD was performed continuously in rats after STZ injection until the end of the experiments. As a comparison, the general maintenance diets were conducted in the control group without the STZ injection. All 40 rats received a surgical procedure at raising 8 weeks.

### Surgery, DO, and postoperative procedures

**Fig. 1:**
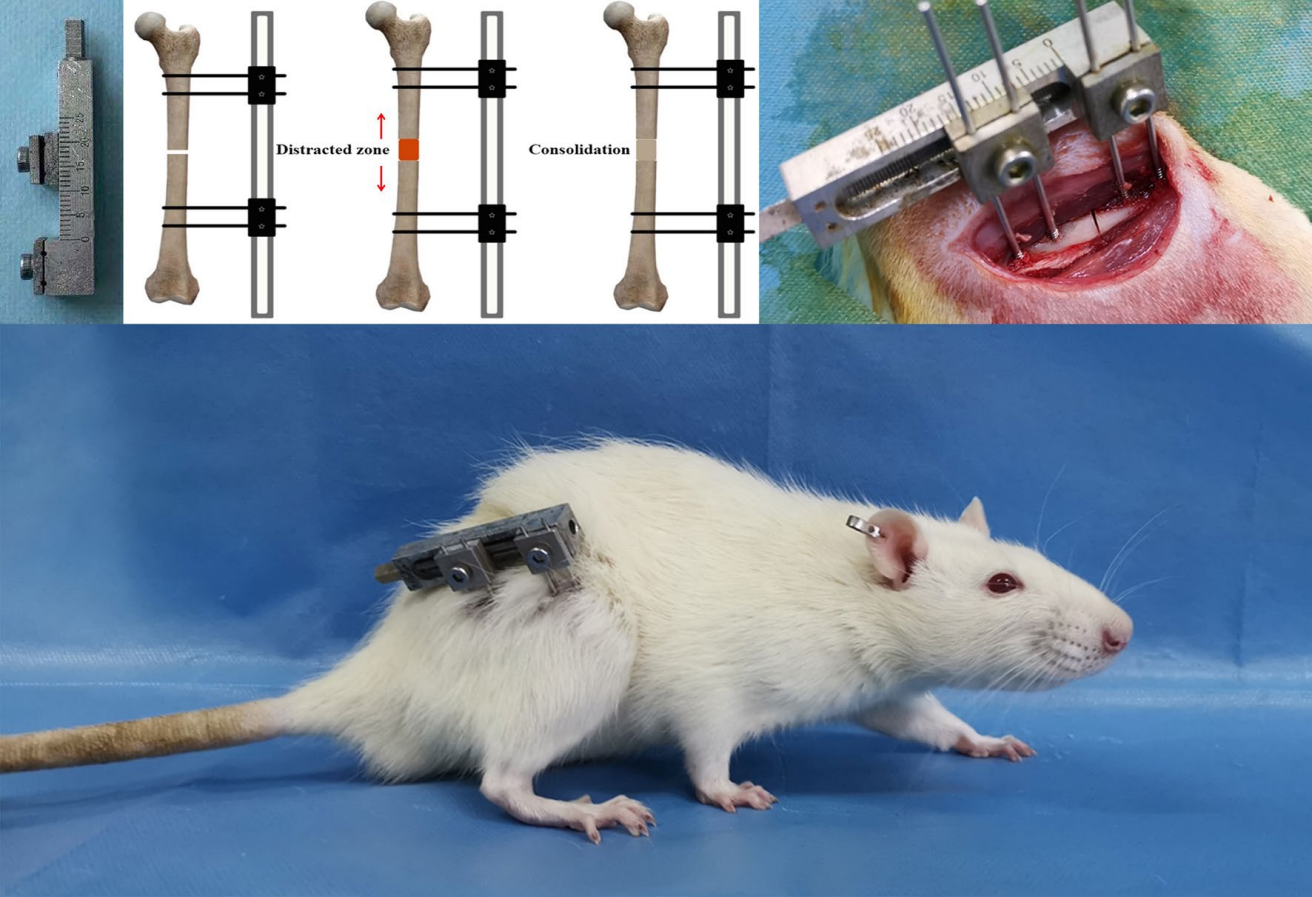
The surgical procedures for the rat right femur model of DO, and the postoperative appearance

In the process, the same team of highly skilled surgeons performed all surgical operations and postoperative procedures. A surgical operation was performed in the eight weeks of raising 40 rats. During the operation, each rat was anesthetized with 2% pentobarbital sodium (3 mg/100 g). In order to prevent infection, benzylpenicillin was administered preoperatively. Under sterile conditions, a monolateral distraction external fixator (designed and manufactured by this research team) was installed on the right femur by using four stainless steel self-tapping screws in the rat, and then a mid-diaphysis transverse osteotomy was performed with the miniature bone saw (Fig. 1) [17]. As the external fixator is adjusted, the proximal and distal ends of the fracture will be separated, indicating a complete osteotomy.

**Fig. 2:**
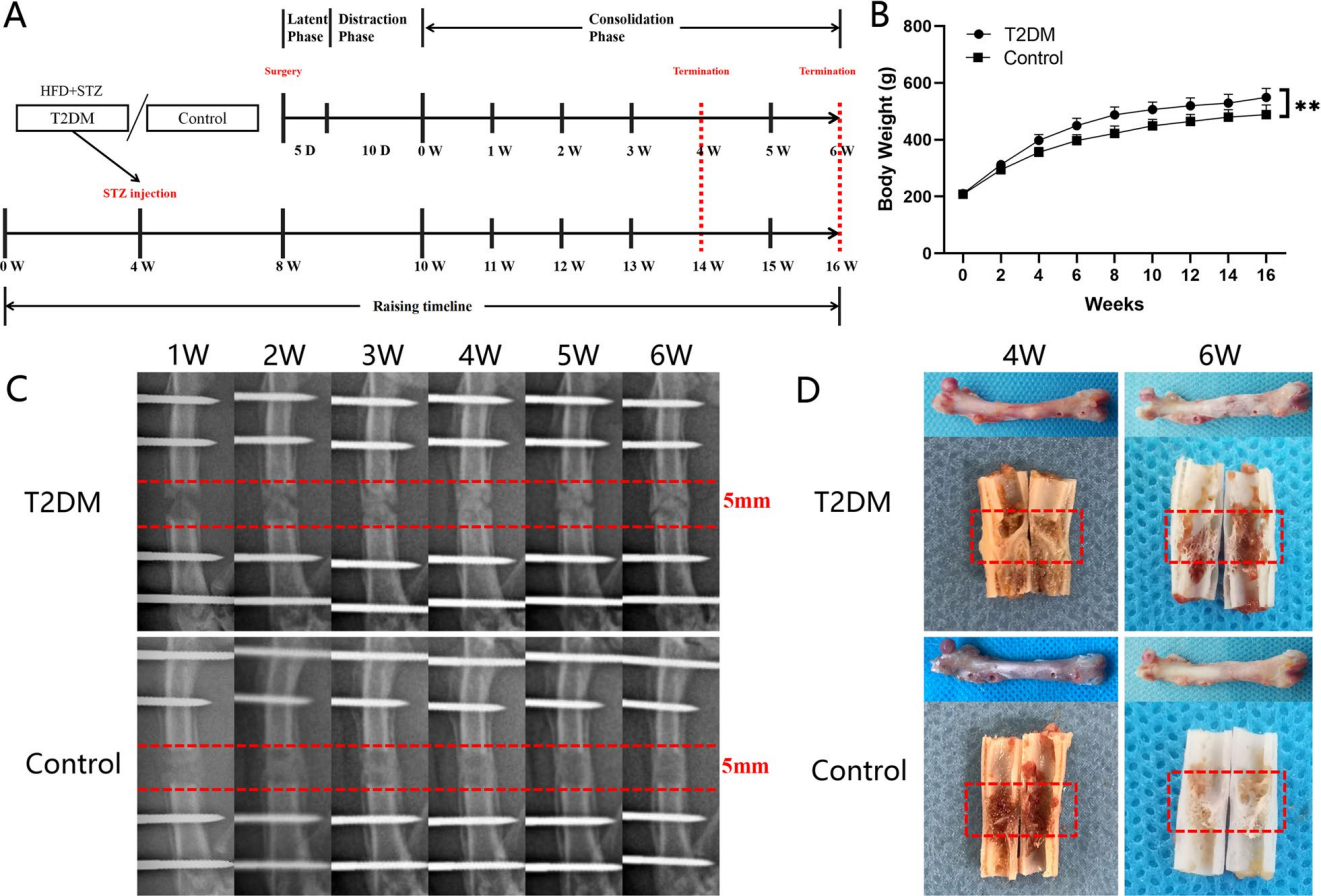
Experimental procedure and observation. **A** Animal raising and surgical observation timeline; **B** The changes in body weight between the two groups; **C** There is a six-week consolidation duration for the distraction X-ray images that regenerate each week; **D** After four- and six-week consolidation, a general image of the specimens can be seen. (*P < 0.05, **P < 0.01)

Postoperative care and further experiments were administered based on the same standardized procedures. Using the antibiotic solution, daily care was performed for the pin sites. Following surgery, laboratory rats received intramuscular injections of benzylpenicillin every day for three days to prevent infection. During the experiment, each rat was housed in its cage and allowed to roam freely. Water and food were provided for free. All rats received a DO procedure with three phases [17, 18]: a latency phase (5 days), an active lengthening phase (10 days, 0.25 mm/12 h), and a consolidation phase (28 or 42 days). Four and six weeks (28 and 42 days) after consolidation, sacrificial rats were randomly selected, and bone samples were harvested from both femurs for further experiments (*n* = 10 per group) (Fig. 2A).

### Digital radiographic analysis

Digital radiographic analysis was performed to evaluate bone regeneration of the distraction zone in each rat. After isoflurane anesthesia, each rat was subjected to an anteroposterior (AP) radiographic examination weekly until sacrifice with the same digital radiographic apparatus (HF400VA, MIKASA X-RAY Co., Ltd., Tokyo, Japan) and conditions (44 kV, 4.5mAs). According to the radiological results, bone consolidation was manifested as the callus and fracture lines disappear, and trabeculae develop through the fractured area in the distracted segments.

### Micro-computed tomography (CT) analysis

In order to evaluate the microstructural change of bone regeneration in the distraction zone, a quantitative assessment was administrated on the representative femur specimens that were collected at the 6 weeks of consolidation (*n* = 3 per group) by using micro-CT imaging (80 kV, 313 μA for 0.203 s, voxel size 18 μm; SkyScan 1176, Bruker, America). Skyscan NRecon software was used to optimize and recompute the scanned images, and Skyscan CTAn software was used for three-dimensional (3D) analysis based on the manufacturer’s instructions. According to a previous study, the region of interest (ROI) was defined as the distraction zone surrounded by the outlined periosteum from the proximal to distal ends [19]. In ROI, bone consolidation was manifested as cortical bone reconstruction and complete recanalization of the marrow cavity. Additionally, bone mineral density (BMD) and bone volume/total tissue volume (BV/TV) measurements were measured on bone tissues within the ROI.

### Biomechanical test

The strength of regenerated bone tissue was evaluated using mechanical properties (*n* = 3 per group). In this procedure, a three-point bending test (RGM-3005 T, ShenZhen Reger Instrument Co., Ltd., China) was administrated to assess the samples of 6 weeks of consolidation without external fixators and screws. The unoperated femurs were collected as the control samples in each rat. The long axis of the femur was perpendicular to the blades in this experiment when the span was 18 mm. An average speed of 0.5 mm/min was applied continuously in the distraction zone with the AP direction until failure was achieved. Both healthy and damaged femurs were measured for ultimate load, modulus of elasticity (E-modulus), energy to failure, and stiffness.

### Histomorphometry in non-decalcified tissue

To further analyze the specimens, a 10% formalin buffer solution was applied for 48 h, followed by a 75% ethanol solution. Following termination at each time point, the specimens for each group (*n* = 3) were successively dehydrated and fattened with xylene and then embedded in methyl methacrylate. With the help of a hard tissue microtome, section 10 μm thick were cut. The histomorphometric appearance was observed by a series of tissue stains, including von Kossa, Masson trichrome, Goldner trichrome, and safranin o staining.

### Immunohistochemistry in decalcified tissue

A standard protocol involved deparaffinized the specimens in xylene, rehydrating them in gradient alcohol, and examining them with immunohistochemistry. 0.3% hydrogen peroxide was used to quench endogenous peroxidase activity for twenty minutes. An antigen retrieval solution of 0.4% pepsin was used for 25 min at 37 °C, followed by a blocking solution containing 5% goat serum for 30 min at 37 °C. Subsequently, anti-hypoxia-inducible factor 1α (anti-HIF-1α) (ab216842, Abcam, Cambridge, UK) and anti-vascular endothelial growth factor (anti-VEGF) (sc7269, Santa Cruz, CA, USA) primary antibodies were incubated overnight at 4 °C on sections. After the samples were incubated in a secondary antibody (PV6000, ZSGB-BIO, Beijing, China) for 1 h at 37 °C, a horseradish peroxidase-streptavidin system (ZLI-9019, ZSGB-BIO, Beijing, China) was used to detect the signals, followed by hematoxylin counterstaining. Each section was analyzed with three ROI fields chosen at random and observed at a 200 × magnification. Image Pro Plus 6.0 software was used to analyze the positively stained areas semi-quantitatively.

### Statistical analysis

SPSS 26.0 was performed for statistical analysis. A three-time calculation was performed under the same conditions for each dataset to be analyzed. All continuous variables have been expressed as mean ± standard deviation (SD) throughout this paper. In order to determine the normality of the data, an analysis of the Shapiro–Wilk test was conducted. The statistical differences between two specific groups were evaluated using the independent-samples t-test or the Mann–Whitney U test. A statistically significant difference was considered as *P* < 0.05. Graphs were created using GraphPad Prism v.6.0.

## Results

All rats recovered from surgery and survived until the end of the experiments in these experiments without dying. There were no significant difficulties with daily activities for any of the rats, as they all achieved normal ambulation. All rats in the T2DM group presented an applicable FGL (> 16.7 mmol/L) and achieved the next experiments. In this study, the body weight was collected weekly, and the result showed that the increased body weight was presented in the T2DM group in comparison with control group (Fig. 2B).

### Sequential digital radiographs

**Fig. 3:**
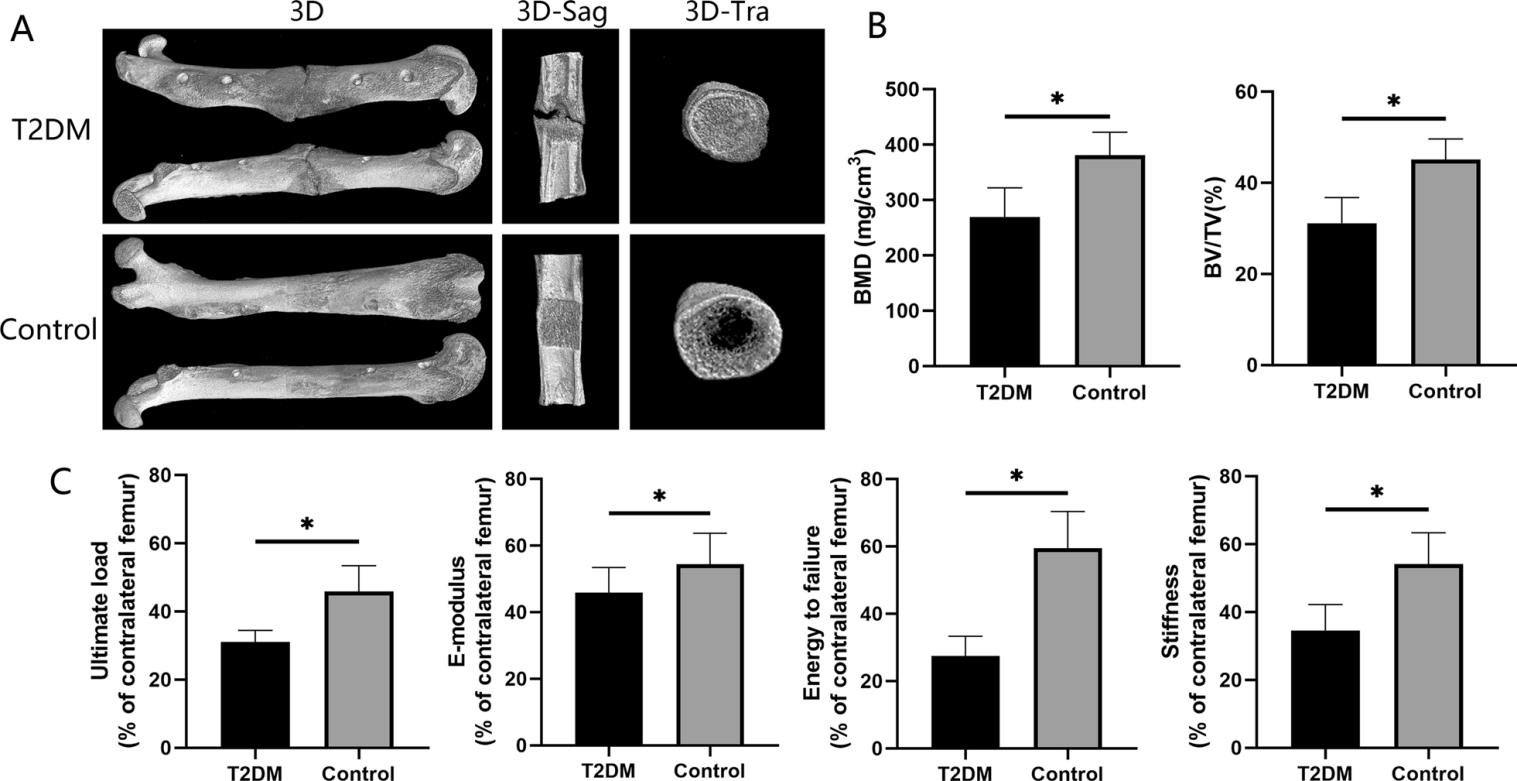
Results of micro-CT evaluation and biomechanics. **A** Representative three-dimensional (3D) micro-CT images of the distraction zone at the termination of the 6-week consolidation; **B** Quantitative evaluation of BMD and BV/TV; **C** Results of mechanical properties and values were normalized to the contralateral femur. (*P < 0.05)

The progress of the bone regeneration and consolidation in distracted segments was monitored weekly using digital radiographs (Fig. 2C). In both groups, newly regenerated callus (early signs of bone reconstruction) was visible in the distracted zone, the bone density was increased gradually over time, and the fracture line was shallowed gradually. In the consolidation phase, there were no significant differences between the two groups regarding bone formation during the first two weeks. Nevertheless, bone regeneration in the T2DM group was slower than in the control group after consolidation for three weeks. By comparing the observed results at four and five weeks, a significant slowdown of bone regeneration was presented in the T2DM group. Additionally, in the distraction zone, there was a significant gap in the T2DM group after 6 weeks of consolidation, while the fracture ends remained unhealed at proximal and distal levels. However, in the control group, this phenomenon did not occur, and bone union occurred after 6 weeks. Similarly, the same results were also shown in the general examination of dissected specimens and micro-CT examination after six weeks of consolidation (Figs. 2D and 3A).

### Three-dimensional (3D) microstructure of bone regeneration

Following 6 weeks of consolidation, the representative micro-CT images revealed that the marrow cavity of the control group was almost completely remodeled. However, a narrow closure of the bone marrow cavity was observed in the T2DM group (Fig. 3A). Additionally, the quantitative results demonstrated that the T2DM group experienced a significant decrease in BMD (269.39 ± 52.32 mg/cm^3^) compared to the control group (380.87 ± 41.44 mg/cm^3^) (*P* < 0.05) (Fig. 3B). Similarly, the BV/TV was also decreased significantly in the T2DM group (31.10 ± 5.70%) compared with the control group (45.14 ± 4.50%) (*P* < 0.05) (Fig. 3B). According to the results, T2DM delayed bone regeneration in DO seriously.

### Mechanical properties of regenerated bone

Following 6 weeks of consolidation, the collected samples were performed to evaluate the mechanical properties using a three-point bending test. The results demonstrated that unsatisfactory outcomes were presented in T2DM group with a decreased ultimate load (31.01 ± 3.39%), E-modulus (34.44 ± 5.06%), energy to failure (27.42 ± 5.87%), and stiffness (34.55 ± 7.66%) than the control group (45.85 ± 7.61%, 54.38 ± 9.33%, 59.41 ± 10.96%, and 54.07 ± 9.30%, respectively) (*P* < 0.05, Fig. 3C).

### Histomorphometry in non-decalcified samples

**Fig. 4:**
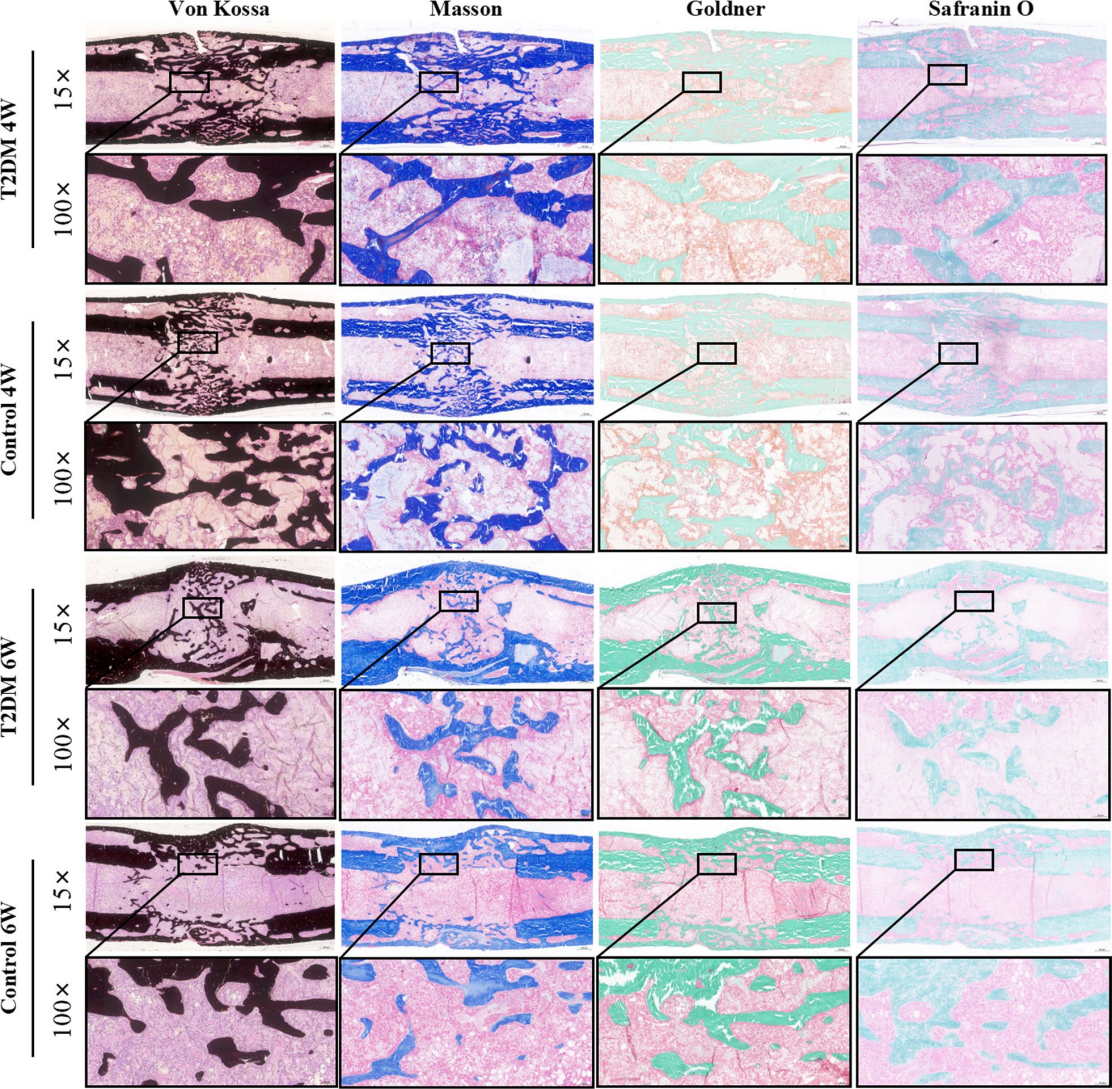
Histomorphological analysis of bone regeneration during the consolidation period. Von Kossa, Masson, Goldner trichrome, and safranin O & fast green staining indicated the decreased bone regeneration in the T2DM group

Histomorphological characteristics of the regenerated bone were assessed in non-decalcified samples, including von Kossa, Masson, Goldner trichrome, safranin O & fast green. Von Kossa staining showed that there was a large amount of calcium in the dark area. Hence, in both of the two groups, the apparent gaps were found in the interested area after 4 weeks of bony consolidation based on von Kossa staining. However, in the control group, newly regenerated calluses were of higher quality and volume. At 6 weeks, the bone marrow cavity was reconstructed and recanalized completely in the control group. However, the phenomenon was not found in the T2DM group. Similarly, according to safranin O, Goldner trichrome, and Masson staining, the observed results demonstrated that T2DM significantly slows bone formation in DO (Fig. 4).

### Histological assessments in decalcified samples

**Fig. 5:**
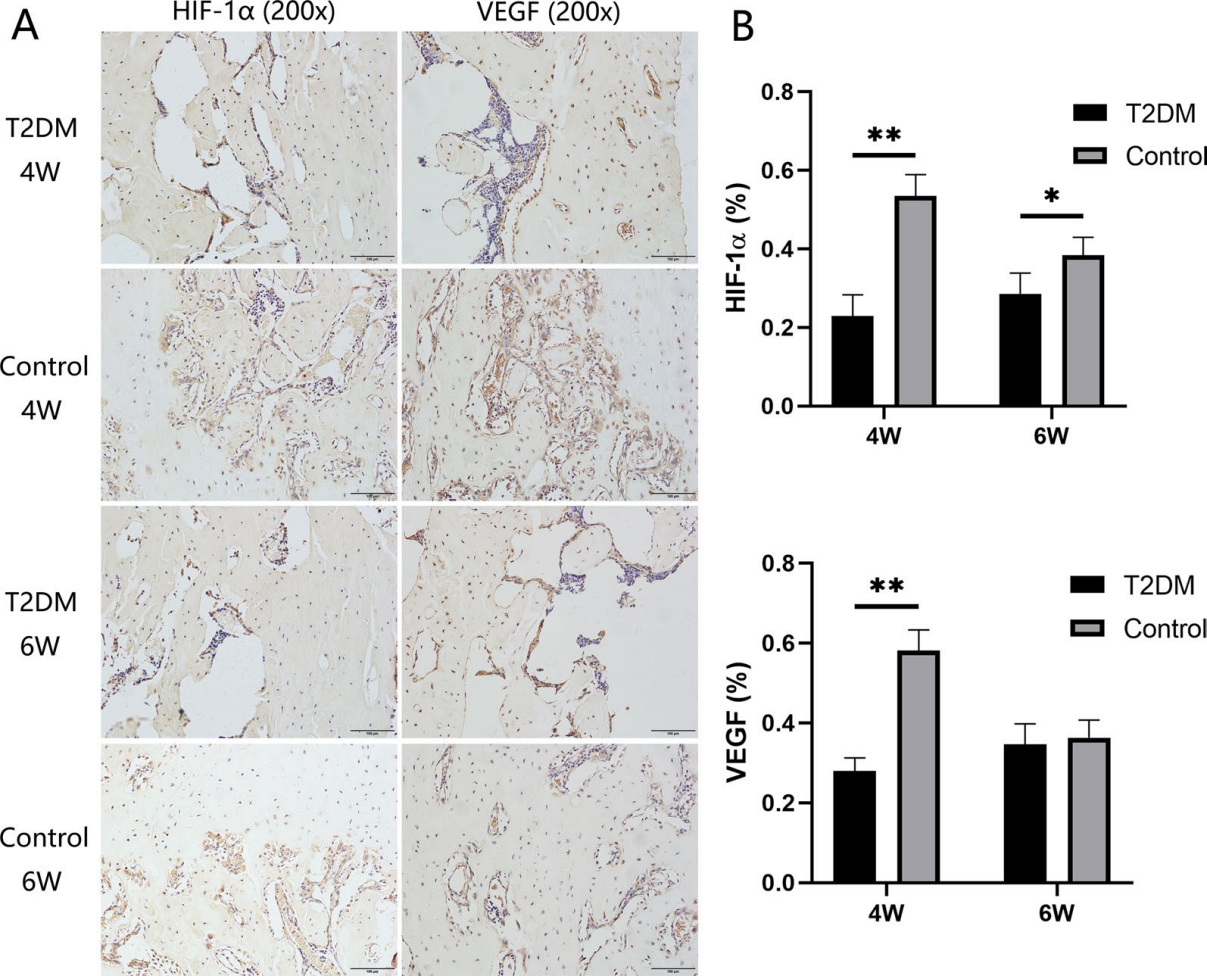
Immunohistochemistry images of HIF-1α and VEGF in the two groups at the termination of 4-week and 6-week consolidation. (*P < 0.05, **P < 0.01)

In the immunohistochemical analysis, the expression of HIF-1α and VEGF was decreased in the T2DM group at 4 weeks of consolidation compared with the control group (*P* < 0.01). Interestingly, the differences between the two groups in the aforementioned indicators decreased at the end of the six-week consolidation period (Fig. 5).

## Discussion

As a result of reports in previous studies on hyperglycemia and bone health [3–9], it has been difficult to assess the consequences of diabetes on bone consolidation and biomechanics in newly regenerated bone. In the present study, the regeneration of the femoral shaft and the bone biomechanics were compared to evaluate the effects of T2DM on bone reconstruction. The results demonstrated that decreased bone regeneration and biomechanics were presented in the distraction gap of the T2DM group.

Currently, several studies suggest that diabetes causes osteoporosis and increases the risk of fractures, regardless of T1DM or T2DM [20–22]. Similarly, a meta-analysis reported that patients with diabetes (T1DM or T2DM) suffered an increased risk for fractures compared to those without a history of diabetes [23]. Generally, the risk was higher in patients with T1DM compared to T2DM according to previous studies [20, 23]. According to a prospective study, compared with the T2DM population and nondiabetic population, T1DM patients suffered a 2.5-fold and sixfold higher incidence of hip fractures, respectively [24]. Though patients with T2DM appear a lower risk of fractures than those with T1DM, the incidence of T2DM is significantly higher than T1DM in the world. Additionally, according to numerous studies, T2DM has been shown to produce a large number of metabolites that might seriously impact tissue regeneration subsequent to injury, including PKC, AGEs, and reactive oxygen species (ROS) [6, 7, 25, 26]. Hence, it is necessary to examine regeneration and biomechanical properties of reconstructed bone tissue under hyperglycemic conditions caused by T2DM.

Our study found that the biomechanics of regenerated bone in the T2DM group was decreased compared with the control group, including ultimate load, E-modulus, energy to failure, and stiffness. This result was consistent with previous studies that demonstrated the decreased biomechanics and increased fragility in the bone of diabetic rat [27–29]. Furthermore, the study showed that diabetes might have a more significant adverse effect on the newly regenerated bone than normal bone in the biomechanical performance, according to the ratio of the surgical and nonsurgical femur. In surgical femur, a series of observations at 4 and 6 weeks of consolidation showed that that bone mass, bone regeneration, and bone mineralization decreased in the T2DM group. The histological results showed that bone regeneration was delayed significantly in the T2DM group, and in the control group at 4 weeks of consolidation, the regenerated bone trabeculae resembled those of the T2DM group at 6 weeks of consolidation. According to the digital radiograph, bone quality, as determined by the volume and continuity of newly regenerated bone, of the T2DM group was inferior to the control group after six weeks of consolidation. Similarly, the micro-CT examination also confirmed that the bone regeneration and recanalization of the medullary cavity were significantly deteriorated in the hyperglycemic condition. The quantitative results concluded that T2DM clearly impaired bone quality by decreasing BMD and BV/TV in regenerated segments. Additionally, a decreased bone regeneration in the T2DM group was also confirmed in the histomorphological assessment compared with the control group. In DO, T2DM had detrimental effects on bone regeneration and biomechanics, as evidenced by the aforementioned compelling findings.

There were various studies focusing on bone strength and diabetes. A recent study showed that the Zucker diabetic fatty rats suffered a decreased bone strength with the lower bone volume fraction (BV/TV) in the neck and shaft of the femur, compared to Zucker lean rats [30]. Additionally, in trabecular bone, a study indicated that diabetic rats had significantly lower vertebral trabecular bone volume and thickness than nondiabetic rats [31]. Furthermore, there was no significant difference in BMD between T2DM rats and nondiabetes rats, according to previous studies [30, 32–34]. However, our study found that the rats with T2DM suffered a decreased BMD in the distracted segments. The result demonstrated that diabetes may have a more serious impact on newly regenerated bone, compared to normal bone. The biomechanical results also confirmed this phenomenon. Compared to the nonsurgical femur, the decline in biomechanics was higher in the surgical femur. The reason for this phenomenon may be closely associated with an unsatisfactory bone regeneration.

It is well known that adequate blood supply plays an important role in bone regeneration and healing. In order to investigate the effect of diabetes on the angiogenesis of the regenerated bone, hypoxia-inducible factor-1α (HIF-1α) and vascular endothelial growth factor (VEGF) immunohistochemical expressions were compared in diabetic rats and nondiabetic rats in the study. The results showed that the decreased expressions of HIF-1α and VEGF were presented in the T2DM group after 4 weeks of consolidation, compared with the control group. However, the differences in expression between the two groups were decreased according to the results after 6 weeks of consolidation, and there was no significant difference in VEGF expression. Hence, we speculated that in comparison with the T2DM group, decreased angiogenesis-related factors and proteins were produced in the more mature regenerate trabeculae in the control group at 6-week consolidation.

As an important factor mediating the adaptive response of the cell to hypoxia, the regulation of HIF activity is heavily dependent on the degradation of the *α* subunit in normoxia [35]. However, numerous studies demonstrated that the hypoxia response was impaired in all tissues in diabetic animals and patients [22–25]. In hyperglycemic conditions, the HIF stability and function were directly repressed at multiple levels, which may be associated with the excessive release of cytokines and proinflammatory factors due to excessive oxidative stress [36–39]. In addition, extensive research has been conducted on the role of the HIF pathway during bone regeneration in regulating osteogenic-angiogenic coupling and confirmed that an important step in bone formation occurs when blood vessels invade avascular cartilage under the influence of VEGF [40–43]. Research has shown that the HIF-1α and VEGF signaling triggered by tissue hypoxia would induce angiogenesis and osteogenesis [40]. However, in a hyperglycemic condition of T2DM, the excessive oxidative stress was activated, and a lot of ROS and downstream proinflammatory factors were released, which may suppress the activity of the HIF pathway and decreased bone formation [35, 37–39]. Similarly, the study found that a suppressed expression of HIF-1α was presented in diabetic rats, compared with nondiabetic rats.

According to the study, it can be inferred that patients with diabetes may experience a delay in bone regeneration after trauma, which complicates clinical assessment of bone healing time. Additionally, it is important to modify the early weight bearing in diabetic patients further due to the biomechanics of bone healing that are worse in diabetics compared to nondiabetics. Although our study yielded promising results, it also had several limitations. First of all, there was a preliminary study that explored the effect of diabetes on the newly regenerated bone, the specific mechanism behind it is still future investigated. In addition, compared to humans, rats walk on four limbs and receive less weight-bearing and mechanical stimulation on their femurs. Therefore, it is necessary to develop and optimize further experimental methods in order to avoid this difference from occurring. Moreover, a study of the histological and morphological characteristics of the regenerated bone was conducted to evaluate the change of regeneration. Future studies may focus on the molecular mechanisms controlling regeneration. In summary, the observed results demonstrated that diabetes causes undesirable bone regeneration and biomechanics, which may be associated with the suppressed HIF/VEGF pathway under hyperglycemia and oxidative stress.

## Conclusion

Our study demonstrated that T2DM impairs bone health of the reconstructed bone in a rat femoral distraction osteogenesis model, including delaying bone regeneration and decreasing bone biomechanics. Additionally, the study also showed that in hyperglycemia and oxidative stress, the suppressed HIF/VEGF pathway may contribute to the phenomenon. Although the aforementioned limitations are needed to be resolved by further studies, the results suggest that patients with diabetes should be allowed more time to heal their bones and prevent refractures.

## Data Availability

The datasets analyzed during the current study are available from the corresponding author on reasonable request.

## Abbreviations

T1/2DM: Type 1/2 diabetes mellitus
HFD: High fat diet
STZ: Streptozotocin
DO: Distraction osteogenesis
CT: Computed tomography
FGL: Fasting glucose levels
HIF-1α: Hypoxia-inducible factor 1α
VEGF: Vascular endothelial growth factor
PKC: Protein kinase C
AGEs: Advanced glycation end products
ROS: Oxygen species
ROI: Region of interest
BMD: Bone mineral density
BV/TV: Bone volume/total tissue volume
